# The role of e-health literacy and some cognitive factors in adopting protective behaviors of COVID-19 in Khalkhal residents

**DOI:** 10.3389/fpubh.2022.916362

**Published:** 2022-07-22

**Authors:** Hamed Rezakhani Moghaddam, Soheila Ranjbaran, Towhid Babazadeh

**Affiliations:** ^1^Department of Public Health, Khalkhal University of Medical Sciences, Khalkhal, Iran; ^2^Department of Public Health, Sarab Faculty of Medical Sciences, Sarab, Iran

**Keywords:** e-health literacy, health belief model, COVID-19, protective behaviors, Iran

## Abstract

**Background:**

Several vaccines have recently been generated and are being utilized to prevent COVID-19 mortality. Although the disease is causing many fatalities worldwide, preventative practices should be prioritized, even if vaccines are available. Therefore, this study aimed to identify the role of e-health literacy and some cognitive factors in adopting protective behaviors against COVID-19 in Khalkhal residents.

**Methods:**

In the present cross-sectional study we recruited 380 people aged 18–65 according to cluster sampling from September 2021 to December 2021 in Khalkhal County, Iran. Reliable and validated tools were applied to data collection, including the eHealth Literacy Scale (eHEALS) in Persian and the Cognitive factors assessment questionnaire based on the Health Belief Model (HBM). Data were analyzed using Chi-square, one-way ANOVA, independent samples *t*-test, and bivariate correlation. The predictors were also determined using hierarchical linear regression analysis.

**Results:**

The average age of the participants was 35.26 ± 11.51 years. The regression analysis implied that gender (*p-*value = 0.032), education level (*p-*value = 0.001), occupational status (*p-*value = 0.002), income (*p-*value = 0.001), and marriage (*p-*value = 0.001) had statistically significant associations with e-HL. Additionally, education level (*p-*value = 0.001), occupational status (*p-*value = 0.001), income (*p-*value = 0.001), and marriage (*p-*value = 0.002) revealed statistically significant associations with COVID-19 preventive behaviors. Approximately 16.5% of the variation in the COVID-19 protective behaviors is explained by the cognitive factors and the demographic variables. Overall, demographic, cognitive, and e-HL variables were able to explain roughly 35.5% of the variation in COVID-19 protective behaviors. Furthermore, self-efficacy was the strongest predictor of protective behaviors (β = 0.214).

**Conclusions:**

HBM constructs successfully predicted the role of e-health literacy and some cognitive factors in adopting COVID-19 protective behaviors. People with high socioeconomic levels were better at e-health literacy and COVID-19 protective behaviors during the pandemic. Moreover, applying approaches to adopting COVID-19 protective behaviors is essential, especially in low socioeconomic status (SES) groups.

## Introduction

Coronaviruses are a vast family of viruses and subcategories of coronaviruses that can cause everything from a typical cold to more serious disorders (1). Coronaviruses have been among the pathogens of the upper respiratory tract for a long time, accounting for 10–30% of common colds. Four types of human coronavirus have been found so far, namely, Hcov229E, Hcovoc43, HcovNL63, and HcovHKu1. However, only three of these viruses have caused severe disease and epidemics: SARS in China in 2002, which killed 800 people, and MERS (Middle East Respiratory Syndrome) in Saudi Arabia in 2012, which killed 858 people in the Middle East (2). However, the family's most recent virus, called n-COVID-19, was discovered in Wuhan, China, in December 2019 and caused significant morbidity and mortality (3). It has already spawned one of the world's most significant pandemics, deemed a global public health emergency. COVID-19 is an acute respiratory infection related to the coronavirus SARS (4).

COVID-19 outbreaks can be avoided by taking preventative steps and practicing self-care. Self-care is a health-promoting behavior, and people who practice it place a high value on their health (5). Participation and acceptance of responsibility on the part of the individual to prevent the occurrence of diseases in himself is an essential component of self-care. Self-care enables the patient to play an active role in their care and treatment and to take responsibility for their own care. Self-care is an action in which a person uses their knowledge, skills, and abilities to independently improve their health status (6).

According to Weber, a person's lifestyle is influenced by his or her social position. When it comes to health-related behaviors, collective patterns of health-related behaviors rely on the selection of options available to them based on the probability of living; these probabilities of living include age, gender, and other appropriate structural variables, which influence lifestyle choices (self-care) (5).

In a study titled “Self-care behaviors in patients with heart failure,” Shojaei et al. (7) concluded that self-care habits are beneficial. Men have more appropriate care for their behaviors than women, and self-care habits are inversely and strongly connected to age. People with a high education have better judgment and decision-making skills to manage their actions, according to the Rockwell study (8).

Because the virus has a lipid coating (3), the best way to kill it is to wash your hands frequently and avoid contacting contaminated surfaces.

Some of the preventive methods against this disease include disinfecting with 70% alcohol, using masks and gloves, only leaving the house for needed duties, and avoiding contact with sick persons (9). Traffic restrictions in urban areas, closure of universities, schools, and markets, cancellation of flights, screening of people by health personnel, home quarantine, and disinfection of premises and environment are among the policies adopted by the country to prevent and slow the COVID-19 epidemic's upward trend in Iran (10).

Some significant reasons, such as the rise in chronic diseases and online access to health information, have recently raised the demand for individual participation in decision-making and health management, as well as the importance of health literacy (11). Health literacy is defined as an individuals' ability to acquire, interpret, and utilize basic health information is critical for selecting whether or not to adopt health habits (12). According to another definition, health literacy is a collection of skills that includes reading, listening, analysis, decision-making, and the ability to apply these skills to health problems, and is not necessarily tied to years of school or general reading proficiency (13). Resources such as media, counseling, publications, and educational posters should be used to improve health literacy in society (14). Health and lifestyle can be improved by having easy access to health information and education in simple language (15). Health literacy is now a global topic and debate (16), and policymakers are considering it as one of the essential tools to enhance society's health and improve the quality of health care services because of its important role in how individuals make decisions in health-related domains (17).

In critical situations such as epidemic disease, active participation of people, and adherence to hygiene and self-care is one of the strengths to deal with that disease or crisis properly. Increasing awareness and avoiding false stress by giving the right information to people through the media, as well as increasing health literacy, can be effective factors in adopting health-promoting behaviors (18). A study by Tentine Sentell et al. (19) highlights the importance of health literacy in emerging decision-making networks. People with strong health literacy engaged in more preventative behaviors than those with low health literacy, according to Scoot et al. (20).

E-health literacy is defined as the ability to find, comprehend, and evaluate health-related information from electronic sources and apply that information to solve or detect a health problem (21). This concept incorporates two key components: individuals' ability to (1) comprehend health information and (2) make appropriate decisions based on that information. E-health literacy highlights the expanding role of information and communication technology in health information and is based on the notion of health literacy (22). E-health literacy necessitates knowledge of health, information, media, computers, and the internet (23).

The skills and information that shape e-health literacy are continually shifting due to the rapid evolution of these technologies. Health literacy and e-health literacy have been highlighted in the twenty-first century as global issues (24). Over the next 10 years, the US government aims to spend more than $37 million on health information technology upkeep to make these resources actively available to internet users. A high proportion of internet users browse the internet for health information, according to a study by Fox and Duggan (25). It's also been stated that 9 out of 10 internet users in South Korea look for online health information, while 66% of persons in Europe do so (26).

Given the socioeconomic implications of COVID-19 on all aspects of people's lives, as well as the role of health behaviors in preventing coronavirus and its impact on health information and people's beliefs (27), we decided to conduct preliminary research in this area in 2021 to investigate the role of e-health literacy and some cognitive factors in adopting COVID-19 protective behaviors in Khalkhal residents.

## Methods

### Study setting and subjects

The present cross-sectional study was conducted from September 2021 to December 2021 in Khalkhal County, Ardabil Province, Iran. Khalkhal is in the Ardabil province's southwestern corner, bordering the Gilan province. According to the 2016 census, the county's population was 86,731 people living in 26,779 households. Based on previous studies, 380 people were chosen as a sample using Cochran's formula and cluster sampling (28).

### Data collection

The statistical population included all people in Khalkhal county between the ages of 18 and 65. In the first phase, all three urban health centers in Khalkhal county were considered three clusters. Then the number of necessary samples was randomly selected from all three health centers based on the calculated sample size.

In other words, all families with persons aged 18–65 were plucked according to the census office by referring to each health center, and then a random sampling method was used among them. Participants were contacted after getting permission from the appropriate center to complete the questionnaire, and if they consented to participate in the study, they were requested to complete the questionnaire. Interviews were used to complete the questionnaire for illiterate people.

The inclusion criteria were being 18–65 years old, consent to participate in the study, no psychological problems, and ability to answer questionnaires. Incomplete questionnaires, dissatisfaction to participate in the study, a history of psychiatric hospitalization, or referral to a therapist owing to mental illnesses were all considered exclusion criteria.

### Study instruments and their validity and reliability

The socio-demographic information questionnaire, the eHealth Literacy Scale (eHEALS), and the cognitive factors assessment questionnaire were used to collect data.

### Socio-demographic information questionnaire

Age, gender, marital status, degree of education, employment position, and health information sources were all included in the demographic information questionnaire. Questions like “Do you utilize personal protective equipment (masks, gloves, disinfection, etc.)?” were asked in this section.

### eHealth Literacy Scale (eHEALS)

The appropriate reliability of the Persian translation of the eHEALS was shown in a study by Bazm et al. with Cronbach's alpha = 0.86 (29). This questionnaire comprises eight questions that assess a set of skills required to use the internet for health promotion. A five-point Likert scale was utilized for each item (very poor, poor, average, good, and very good with a score of 1–5).

### The cognitive factors assessment questionnaire

Sedigheh Salavati et al. have validated this questionnaire in the Iranian population (Cronbach's alpha = 0.87) (30). The questionnaire consists of six questions about perceived threats, each of which is rated on a Likert 5-point scale (strongly disagree, disagree, have no opinion, agree, and highly agree), which was scored from 0 to 4. There are also questions about perceived benefits (5 questions), perceived barriers (3 questions), perceived self-efficacy (6 questions), and action cues (3 questions). Five items in the last section of the questionnaire were designed to measure behaviors that prevent coronavirus infection, and they are scored on a scale of 0–4 (always, sometimes, sometimes, rarely, and never).

### Statistical analysis

SPSS software version 21.0 was used to analyze the data, which included descriptive statistics [*n* (%)], Chi-square, one-way ANOVA, and correlation. The predictors were also determined using hierarchical linear regression analysis. A *P-*value of < 0.05 was judged significant.

## Results

Table 1 shows the demographic characteristics of the participants. The participants' average age was 35.26 ± 11.51. The majority of the participants (63.77%) were female and had a diploma level of education (35.8%). Nearly 60% of those who took part in the survey said they were unemployed. Gender (*p-*value = 0.032), education level (*p-*value = 0.001), occupational status (*p-*value = 0.002), income (*p-*value = 0.001), and marriage (*p-*value = 0.001) all had statistically significant associations with eHL. Additionally, education level (*p-*value = 0.001), occupational status (*p-*value = 0.001), income (*p*-value = 0.001), and marriage (*p*-value = 0.002) all revealed statistically significant associations with COVID-19 preventive behaviors.

**Table 1:** Relationship between eHL, HPBs, and some of demographic characteristics among people.

**Variables**		***N* (%)**	**H**	***p*-value**	**HPB**	***p*-value**
			**Me ±SD**		**Me ±SD**	
Gender^**^	Male	139 (36.3)	13.84 ± 7.99	0.032	5.58 ± 3.92	0.455
	Female	244 (63.77)	15.59 ± 7.44		5.30 ± 2.96	
Education level^*^	Under diploma	132 (34.5)	11.20 ± 7.67	<0.001	4.33 ± 3.23	<0.001
	Diploma	137 (35.8)	16.30 ± 7.08		4.71 ± 3.52	
	Super diploma & higher	114 (29.8)	17.67 ± 6.68		6.28 ± 2.89	
Occupation^**^	Unemployed	89 (23.2)	15.42 ± 8.31	0.002	5.43 ± 3.91	<0.001
	Housekeeper	99 (25.8)	12.64 ± 8.23		4.16 ± 2.72	
	Employed	195 (50.9)	15.91 ± 6.85		6.02 ± 3.17	
Income (month) ^*^	<100 dollars	142 (37.1)	13.26 ± 8.07	<0.001	4.37 ± 3.29	<0.001
	100–200 dollars	102 (26.6)	15.58 ± 7.91		5.49 ± 3.24	
	More than dollars	139 (36.3)	16.21 ± 6.78		6.40 ± 3.15	
Marriage ^**^	Single	180 (47.0)	17.37 ± 6.47	<0.001	5.95 ± 3.31	0.002
	Married	203 (53.0)	12.81 ± 8.03		4.92 ± 3.45	
**Residence**	Rural	334 (87.2)	15.20 ± 7.59	0.091	5.53 ± 3.29	**0.54**
	Urban	49 (12.8)	13.22 ± 8.16		4.55 ± 3.57	
**Family size**	3 and lower	164 (42.8)	15.16 ± 8.39	0.646	5.27 ± 3.2	**0.501**
	4 and higher	219 (57.2)	14.79 ± 7.11		5.50 ± 3.38	

* p-Value based one-way ANOVA test.

** p-Value based t-independent test.

According to the bivariate correlation, COVID-19 protective behaviors have statistically significant associations with self-efficacy, perceived benefits, and eHL (*p-*value < 0.05) (Table 2).

**Table 2:** Bivariate correlation matrix of the relationship between HBM structures, eHL dimension, and HPBs.

**Variables**	**1**	**2**	**3**	**4**	**5**	**6**	**7**	**Me ±SD**
**1 = Cues to action**	1							34.51 ±28.81
**2 = Risk perception**	0.391**	1						186.31± 67.90
**3 = Self-efficacy**	0.046	0.015	1					5.35 ± 3.84
**4 = Perceived benefits**	0.010	0.095	0.770	1				2.16 ± 2.07
**5 = Perceived barriers**	−0.019	0.020	0.052	0.0314**	1			6.36 ± 3.70
**6 = eHL**	0.166**	0.033	0.028	0.089	0.082	1		14.95 ± 7.68
**7 = COVID-19 protective behaviors**	0.143	0.047	0.426**	0.406**	−0.53	0.285**	1	5.40 ± 3.33

^*^Correlation is significant at the 0.05 level (two-tailed).

COVID-19 protective behaviors were predicted using hierarchical multiple linear regression. Table 3 shows that in block 1, demographic characteristics were significant predictors of COVID-19 preventive actions. Demographic factors explained 16% of the variation in COVID-19 protective behaviors (*F* = 8.886; *p-*value = 0.001), showing that demographic factors account for roughly 16% of the variation in HPBs. The cognitive components were included in Block 2, which explained an extra 16.5% of the variation (*F* = 18.02; *p-*value = 0.001). In addition, we included eHL in block 3, which explained an extra 3% of the variation (*F* = 17.24; *p-*value = 0.001). Overall, demographic, cognitive, and eHL variables were able to explain roughly 35.5% of the variation in COVID-19 protective behaviors.

**Table 3:** Hierarchical linear regression for prediction of COVID-19 protective behaviors through demographic characteristics and eHL.

**Variables**	***ß***	**R2 change**	***F* change**	**SE**	***p-*value**
**Block 1**					
Age	0.127^*^	0.160	8.886	0.014	<0.001
Gender	0.083			0.339	
Education level	0.204^*^			0.148	
Job	0.212^*^			0.063	
Income	0.168^*^			0.135	
Marriage	0.107^*^			0.447	
Residence	0.055			0.484	
Family size	0.338^*^			0.338	
**Block 2**					
Age	0.138	0.165	18.02	0.013	<0.001
Gender	0.048			0.323	
Education level	0.167^*^			0.136	
Job	0.125^*^			0.059	
Income	0.097			0.124	
Marriage	0.138^*^			0.407	
Residence	0.013			0.444	
Family size	0.012			0.307	
Cues to action	0.047			0.006	
Risk perception	0.027			0.002	
Self-efficacy	0.214^*^			0.045	
Perceived benefits	0.296^*^			0.088	
Perceived barriers	−0.075			0.044	
**Block 3**					
Age	0.059	0.030	17.24	0.013	<0.001
Gender	0.066			0.318	
Educational level	0.125^*^			0.136	
Job	0.127^*^			0.057	
Income	0.095			0.121	
Marriage	0.125^*^			0.399	
Residence	0.013			0.435	
Family size	0.020			0.305	
Cues to action	0.021			0.305	
Risk perception	0.010			0.005	
Self-efficacy	0.240^*^			0.002	
Perceived benefits	0.272^*^			0.045	
Perceived barriers	−0.077			0.086	
eHL	0.206^*^			0.043	
Total R2	0.355	–	–	–	–
Adjusted R2	0.330	–	–	–	–

* p < 0.05.

## Discussion

E-health literacy can be essential during lockdown and pandemic situations like COVID-19, where health information is virtually provided through electronic sources, and decision-making plays the main role in reducing infectious diseases spread and adopting health protective behavior. Hence, the present study aimed to examine the role of e-health literacy and some cognitive factors in adopting COVID-19 protective behaviors.

Demographic characteristics including gender, education level, occupational status, income, and marriage had statistically significant associations with e-HL. In this study, e-health literacy is affected by high incomes, high education levels, good occupational status, and being unmarried. These were consistent with the results displayed by Alipour and Payandeh in Iran (31), Zakar et al. in Pakistan (32), Guo et al. in Hong Kong (33), Dadaczynski et al. in Germany (34), Rosário et al. in Portuguese (35) on COVID-19, digital health literacy (DHL) and health information-seeking behaviors of University students except for gender. Various results have been reported in terms of gender in these studies. In this research, the mean score of e-health literacy in women was higher than in men. Similarly, a study reported a high level of digital health literacy (DHL) in female students (32). In contrast, male students had fewer problems than female students in terms of DHL dimensions such as adding self-generated content and evaluating reliability during the COVID-19 pandemic (35). In the other studies, female students had poor DHL among whole dimensions (34). Women are more likely to adopt health protective behavior as compared to males (36, 37) and may be more likely to find, comprehend and evaluate health-related information from electronic sources and apply that information to solve or detect a health problem but this can be different in the cultural and social contexts of regions and countries. According to the results of a study, contextual, environmental, and sociodemographic factors affect e-health literacy (38). The results of the previous study demonstrated low levels of education and income status were barriers to achieving e-health literacy (39–41). People with poor occupational status, low incomes, and low education levels are less likely to have access to electronic information resources and the internet. Hence, these groups of people require particular attention during the COVID-19 pandemic when misinformation and rumors are spread in terms of COVID-19 and it is difficult to distinguish between right and wrong. In order to promote health, given the limited resources of literacy, it is important to identify the characteristics of people who are at lower risk of e-health literacy (42).

Also, education level, occupational status, income, and marriage revealed statistical associations with COVID-19 preventive behaviors. Low levels of education, poor occupational status, low incomes, and being married were less likely to adopt COVID-19 preventive behaviors. This result is similar to the findings of Firouzbakht et al. in Iran (43). Shmueli in Israeli (44), Riiser et al. in Norway (45), and Wolf et al. in the US (46). Guo et al. in Hong Kong (33). Ko et al. in Taiwan (47). People in the lower socioeconomic status (SES) groups have unstable working conditions and incomes, which exacerbate the prevalence of COVID-19 and its consequences (48). Surprisingly, in our study, being unmarried was a determinant factor for e-health literacy and adopting COVID-19 preventive behaviors. A study by Akbarpour et al. found being married was associated with anxiety about the COVID-19 pandemic (49). Married persons may be less likely to use electronic information resources because of their anxiety and fewer opportunities. It is necessary to pay more attention to demographic characteristics at the individual level in order to provide better and equitable health services, especially during the COVID-19 pandemic; because the COVID-19 pandemic disproportionately affects the low socioeconomic status group (SES), people with limited access to health care for COVID-19, living in crowded places where the risk of transmitting the disease is higher and not allowed to work remotely (48).

The results of this research demonstrated COVID-19 protective behaviors have statistically significant associations with self-efficacy, perceived benefits, and e-HL. These results indicated that a high level of self-efficacy, perceived benefits, and e-HL led to high adherence to COVID-19 preventive behaviors. These are consistent with a study conducted in South Korea on COVID-19 infection-preventive behaviors of undergraduate students (50), in Iran on preventative health behaviors from COVID-19 in adults (51). The self-efficacy construct was the strongest determinant of COVID-19 protective behaviors in this study. In the previous studies conducted by Fathian-Dastgerdi et al., Mirzaei et al. self-efficacy construct was found the strongest predictor COVID-19 protective behaviors in adolescents and adults, respectively (52). Also, a study observed consumer self-efficacy was the main factor affecting their continued use of E-wallets (53). In the short term, health threat constructs to adopt protective behaviors of E-wallets during the COVID-19 pandemic, and in the longer term, addressing expected value and benefits and self-efficacy are crucial (53). A high level of self-efficacy has a significant role in overcoming the perceived barriers and leads to adopting COVID-19 preventive behaviors (51). Perceived benefits during the COVID-19 outbreak may persuade individuals to adopt COVID-19 protective behaviors to reduce the dangerous consequences. Given that, individuals are more likely to focus on self-efficacy and perceived benefits during the outbreak of infectious diseases like COVID-19 than perceived barriers, risk perception, and cues to action. This finding can help policy makers and health care providers to address targeted programs, appropriate to needs and avoid threats and fears. Another study showed a negative relationship between perceived susceptibility and COVID-19 protective behaviors in adolescents (52). Also, digital health communication media (DHCM) usage in terms of healthy food information during the COVID-19 pandemic did not associate with the perceived threat of the COVID-19 outbreak (54). During the COVID-19 pandemic, risk perception, perceived barriers, and cues to action factors may predict protective behaviors at the onset of the pandemic, but internal factors such as self-efficacy and behavioral benefits can contribute to continuous health behaviors when the disease leads to death. A study in Australia indicated a low level of health literacy in adults led to a low level of understanding of COVID-19 symptoms, identifying COVID-19 protective behaviors, and difficulty accessing information and understanding messages (55). Health literacy is one of the determinants of COVID-19 health protective behavior (36). Adequate e-health literacy can help to reduce misinformation about COVID-19 because e-health literacy is based on the notion of health literacy (22), and misinformation about the COVID-19 pandemic is associates with a low level of health literacy (55). A study by Sánchez-Arenas et al. in Mexico found a high level of health literacy and availability of COVID-19 information were the main factors in preventive health behavior in adults (56). Individuals with a low level of health literacy are more likely to be at risk for COVID-19 disease, likely to have higher fear and depression, lower health-related quality of life, and less likely to adherence COVID-19 health protective behaviors (37, 45, 57, 58). Adequate e-health literacy promotes people's adherence to COVID-19 health protective behaviors and health guidelines about COVID-19 (33, 59). It may be because individuals with a high level of e-health literacy use more search strategies and can identify better quality health information.

Demographic factors explained 16% of the variation in COVID-19 protective behaviors. The cognitive components explained an extra 16.5% of the variation. In addition, we included e-HL in step 3, which explained an extra 3% of the variation. Overall, demographic, cognitive, and e-HL variables were able to explain roughly 35.5% of the variation in COVID-19 protective behaviors. This model has been successful in determining the role of e-health literacy and some cognitive factors in adopting COVID-19 protective behaviors. This result is consistent with the findings of Shahnazi et al., who implied constructs of the health belief model (HBM), fatalistic beliefs, and demographic factors were determines of COVID-19 protective behaviors (51). This finding is also similar to those reported by Fathian-Dastgerdi et al. in Iran which HBM constructs predicted a 46% variance of COVID-19 preventive behaviors among adolescents (52). Daragmeh et al., also reported Health Belief Model (HBM) and Technology Continuous Theory (TCT) determined a 55.9% variance in individuals' intention in terms of E-wallet usage during the COVID-19 pandemic (53). The study of Jadil et al. implied HBM constructs explained 33.1% of variance COVID-19 preventive behaviors in the whole Moroccan and Indian populations [60]. Similarly, Karimy et al. and Mirzaei et al. demonstrated that 27 and 29.3% of the variance in the COVID-19 preventive behaviors were described by HBM constructs, respectively [61, 62]. A previous study found that HBM and TPB constructs as well as demographic and health-related factors predicted 78% of the variance intention to get the COVID-19 vaccine. Higher levels of perceived benefits COVID-19 vaccine, perceived severity of the COVID-19 pandemic, cues to action, subjective norms, and self-efficacy correlated to intention to receive the vaccine (44). This showed that determinants of intention to get vaccinated against COVID-19 may differ from COVID-19 preventative behaviors.

The results of the present study indicate the need to improve self-efficacy and perceived benefits among adults, especially among those who have low self-efficacy in dealing with the disease or need to evaluate the benefits of adopting COVID-19 protective behaviors.

Given the above, to identify unknown factors affecting human behavior during the pandemic in various societies and cultures, the use of models and theories can be useful. Hence, future studies are suggested to apply the other models and theories such as Fear's theory with a focus on fear, barriers and risk perception constructs.

## Limitations

Given this study was performed in Khalkhal city, Ardabil Province, which is situated in northwestern Iran. Turkish is the common language of the people of this region. Therefore, generalizing the results of this study should be done with caution to other regions of Iran with various cultures, especially Persian-speaking regions.

## Conclusion

The research results revealed high education level, poor occupational and income status, and being married were determinants of e-HL and COVID-19 protective behaviors. Also, the female gender was associated with e-HL. HBM constructs successfully predicted the role of e-health literacy and some cognitive factors in adopting COVID-19 protective behaviors. Perceived self-efficacy was the strongest factor in COVID-19 protective behaviors in adults. It is necessary to address the main factors in adopting COVID-19 protective behaviors, especially sociodemographic, e-HL, and self-efficacy, to promote preventive behaviors during the COVID-19 pandemic. These findings can help policy-makers and health care providers to perform interventions to improve e-HL and self-efficacy with a focus on low socioeconomic status (SES) groups and design and use health messages and platforms appropriate for these groups.

## Data availability statement

The raw data supporting the conclusions of this article will be made available by the authors, without undue reservation.

## Ethics statement

The studies involving human participants were reviewed and approved by Khalkhal Faculty of Medical Sciences (number: IR.KHALUMS.REC.1400.008). The patients/participants provided their written informed consent to participate in this study.

## Author contributions

HR and TB conceptualized and designed the study and drafted the initial manuscript. SR carried out the statistical analysis, drafted the initial manuscript, and reviewed the manuscript. All authors approved the final manuscript as submitted and agree to be accountable for all aspects of the work.

## Conflict of interest

The authors declare that the research was conducted in the absence of any commercial or financial relationships that could be construed as a potential conflict of interest.

## Publisher's note

All claims expressed in this article are solely those of the authors and do not necessarily represent those of their affiliated organizations, or those of the publisher, the editors and the reviewers. Any product that may be evaluated in this article, or claim that may be made by its manufacturer, is not guaranteed or endorsed by the publisher.
